# Supplementary material for: Possible role of caffeine in nicotine use onset among early adolescents: Evidence from the Young Mountaineer Health Study Cohort
Source: PLoS One. 2023 May 11;18(5):e0285682. doi: 10.1371/journal.pone.0285682 (PMC10174549; doi:10.1371/journal.pone.0285682)
Supplement: Attachment — Submitted filename: Response 2 to Reviewers.docx [file pone.0285682.s003.docx]

**Manuscript PONE-D-22-23716R1**

**Reviewer 2, additional comments:**

*Comment 1*: Thank you for inviting me to review the revised manuscript Possible role of caffeine in nicotine use onset among early adolescents: Evidence from the Young Mountaineer Health Study Cohort for consideration in PLOS ONE. The authors have addressed all comments from the previous round of review. However, the authors can still improve on descripting their data collection procedure (see comments below). There are several minor grammatical-type inconsistencies throughout the paper. Since PLOS ONE does not copyedit accepted manuscripts, I would suggest the authors carefully read through the manuscript and make any needed small edits (see comments below for some suggested edits).

*Our Response*: Thank you for taking the time to provide additional comments to improve our work. We greatly appreciate the reviewer’s dedication in helping us provide a quality manuscript for further consideration.

*Comment 2*: Introduction: Line 68 change “(e.g. high-school students)” to (e.g., high-school students).

*Our Response*: The requested change has been made.

*Comments 3-5*: Materials and Methods.

Check throughout the manuscript for 1 vs 2 spaces after periods (see line 98, also line 27 in the abstract).

Line 104 “WV State”, just write out West Virginia

Line 105 change “COVID19” to “COVID-19” to be consistent throughout the paper

*Our Response*: We have carefully checked all double spaces after periods and made the additional requested changes.

*Comments 6-7*: Table 1:

“ENDS only at follow-up” change variable to “ENDS use only at follow-up”

“Coffee 335 (28.27%” missing closing parentheses

*Our Response*: Thank you. Those changes have been made.

*Comments 8-9*: Procedure:

Line 123-124 I am a bit confused about what “designated class-room hours from home” means. Did research staff come into individual homes? Earlier in the manuscript it sounded like all participants were in school at least part time and all others excluded. This phrase made me think maybe I misunderstood what was previously stated.

I don’t think this applies but in case it does: Did the authors check for any differences in responses between students who took the survey at home vs at school? I worry that students taking the survey at home where parents may be present could influence responses.

*Our Response*: We agree with the reviewer that accessing survey participants from home warrants special attention with regards to the potential of outside influences, such as from parents or caregivers. This is precisely what we did and attempted to convey in the narrative. During the COVID-19 pandemic many schools operated solely online for different periods of time. If our data collection was planned to take place while schools were operating solely online, we attempted to minimize potential interference from others (such as parents/caregivers or siblings) by ensuring that the surveys were run during the time of a designated class while the student camera was turned on, as opposed to simply sending a survey link to the student’s email address. This way we preserved a degree of supervision regarding who would be responding to the survey, and control over the time when the survey was responded to by each student. We have revised the sentence pertaining to this issue. It now reads: “Students responded to the surveys either inside schools or during designated online classes from home with their camera turned on, depending on accessibility during COVID-19 mitigation efforts”.

*Comments 10-13*: Results/Discussion:

Line 212-213 You write “ENDS devices” in several places. Just say ENDS and remove the word devices. This is also in the discussion several times. Please check and change throughout the manuscript.

Line 225 “The second model included exclusive ENDS as...” (insert “use”)

Line 242 add the word “use” after ENDS “...we also ran our models to include only ENDS [use] as the outcome variable”

Line 249 “Further, consistent with recent studies, ENDS was” change to “ENDS use was” or make another change. The sentence reads oddly.

*Our Response*: We have carefully reviewed and revised all instances of “ENDS” so that the manuscript now solely states “use of ENDS”, or “ENDS use” and the word “devices” has been removed.

*Comment 14*: Line 253 “...commonly used nicotine product...” change to “...commonly used nicotine products...”

*Our response*: The change has been made.
