# Supplementary material for: Possible role of caffeine in nicotine use onset among early adolescents: Evidence from the Young Mountaineer Health Study Cohort
Source: PLoS One. 2023 May 11;18(5):e0285682. doi: 10.1371/journal.pone.0285682 (PMC10174549; doi:10.1371/journal.pone.0285682)
Supplement: Attachment — Submitted filename: Response 3 to Reviewers.docx [file pone.0285682.s004.docx]

**Manuscript PONE-D-22-23716R2**

**Reviewer 2, additional comments:**

*Comment 1, Editorial*: Please review your reference list to ensure that it is complete and correct. If you have cited papers that have been retracted, please include the rationale for doing so in the manuscript text, or remove these references and replace them with relevant current references. Any changes to the reference list should be mentioned in the rebuttal letter that accompanies your revised manuscript. If you need to cite a retracted article, indicate the article’s retracted status in the References list and also include a citation and full reference for the retraction notice.

*Our Response*: We have carefully reviewed the reference list and made minor changes to four references. No reference was retracted or added.

*Comment 2*: Please provide rationale for recoding caffeine intake into deciles instead of using raw values.

*Our Response*: We have added the following sentence at the end of the description of the caffeine variable (lines 152-153): “Due to the wide range (0 – 3000mg) and high negative skew, the continuous variable was recoded into deciles for simpler interpretation.”

*Comment 3 and Editorial*: Statement at the end of discussion "Conversely, although nicotine products are not all equally harmful to health, no one seriously questions the potential for harm posed by nicotine-containing products in general" is quite an unsupported generalization and should be rephrased or removed. There are many well known and well studied harms of nicotine, independent of the health risks caused by the non-nicotine components of nicotine-containing products.

*Editorial*: As suggested by our referee, the health-related negative effects of nicotine are well known, also in its pure form. Please avoid such sentences.

*Our Response*: We have revised the sentence to read as follows: “Conversely, no one seriously questions the harm posed by nicotine-containing products [41]”.
